# Sufficiency health-wise: sustainable paths towards planetary and public health

**DOI:** 10.3389/fpubh.2024.1497657

**Published:** 2024-11-19

**Authors:** Klaus Geiselhart, Maik Damm, Niklas Jeske, Alexia Knappmann, Gabriela Pen Nasser, Laura Franziska Roth, Regine Unkels, Andrea Sylvia Winkler, Jennyfer Wolf, Timo Falkenberg

**Affiliations:** ^1^Institute of Geography, Friedrich-Alexander-University Erlangen-Nürnberg, Erlangen, Germany; ^2^Global Health Hub Germany, Planetary Health Working Group, Berlin, Germany; ^3^Animal Venomics Lab, Fraunhofer Institute for Molecular Biology and Applied Ecology IME, Giessen, Germany; ^4^LOEWE Centre for Translational Biodiversity Genomics (LOEWE-TBG), Frankfurt am Main, Germany; ^5^Institute for Insect Biotechnology, Justus Liebig University Giessen, Giessen, Germany; ^6^Charité – Universitätsmedizin Berlin, Corporate Member of Freie Universität Berlin and Humboldt-Universität zu Berlin, Institute of General Practice and Family Medicine, Berlin, Germany; ^7^Vivantes Klinikum im Friedrichshain, Berlin, Germany; ^8^Wateraid Germany, Berlin, Germany; ^9^Instituto Melhores Dias, São Paulo, Brazil; ^10^Albert-Ludwigs-Universität Freiburg, Medizinische Fakultät, Freiburg, Germany; ^11^Department of Neurology, TUM University Hospital, Technical University of Munich (TUM), Munich, Germany; ^12^Center for Global Health, School of Medicine and Health, Technical University of Munich (TUM), Munich, Germany; ^13^Consultant Women’s Health and Planetary Health, Bonn, Germany; ^14^Department of Community Medicine and Global Health, Institute of Health and Society, University of Oslo, Oslo, Norway; ^15^Department of Global Health and Social Medicine, Harvard Medical School, Boston, MA, United States; ^16^Consultant in Environment, Climate Change and Health, Freiburg, Germany; ^17^Institute for Hygiene and Public Health, University Hospital Bonn, Bonn, Germany

**Keywords:** structural prevention, sustainability, planetary health, health system-organization and administration, notions of health and wellbeing

## Abstract

Planet Earth is threatened by the human population. Energy and resource use are far beyond the planet’s carrying capacity. Planetary Health suggests an alternative idea of prosperity as the best possible human health for all within planetary boundaries. This implies giving priority to ecology because human health depends ultimately on the integrity of the global biosphere. This paper presents a Health Sufficiency Framework, based on the Doughnut Economics Model. It is meant to fuel discussions on delicate topics of the required transformations of health care and public health.

## Introduction

Planet Earth is on the verge of becoming uninhabitable for many species, including humankind. Due to narrow ideas about wealth, dominant especially among the most economically rich parts of the human population, energy and resource use are far beyond the planet’s carrying capacity. Focusing on Planetary Health (1–3) can help society formulate an alternative idea of prosperity as representing the best possible human health for all within planetary boundaries (4, 5). This implies giving priority to ecology because human health depends ultimately on the integrity of the global biosphere. Focusing on Planetary Health requires restructuring our notion of wealth into a health-related idea of a good life and well-being. It requires diverging from environmentally exploitative and unhealthy consumption-oriented lifestyles perpetuated by growth-dependent economic systems. Transformation must happen in several arenas of change, such as those of wealth and consumption, energy and resource use, mobility, nutrition, urban life, and industrial production, all of which must be addressed under the concept of living within a coherent natural environment (6). Moreover – and this is the main focus of this paper – it requires a transformation in the health systems towards sustainability, prevention and well-being. Providing health care within planetary boundaries cannot be achieved solely by making clinical practice more efficient.

**Figure 1:**
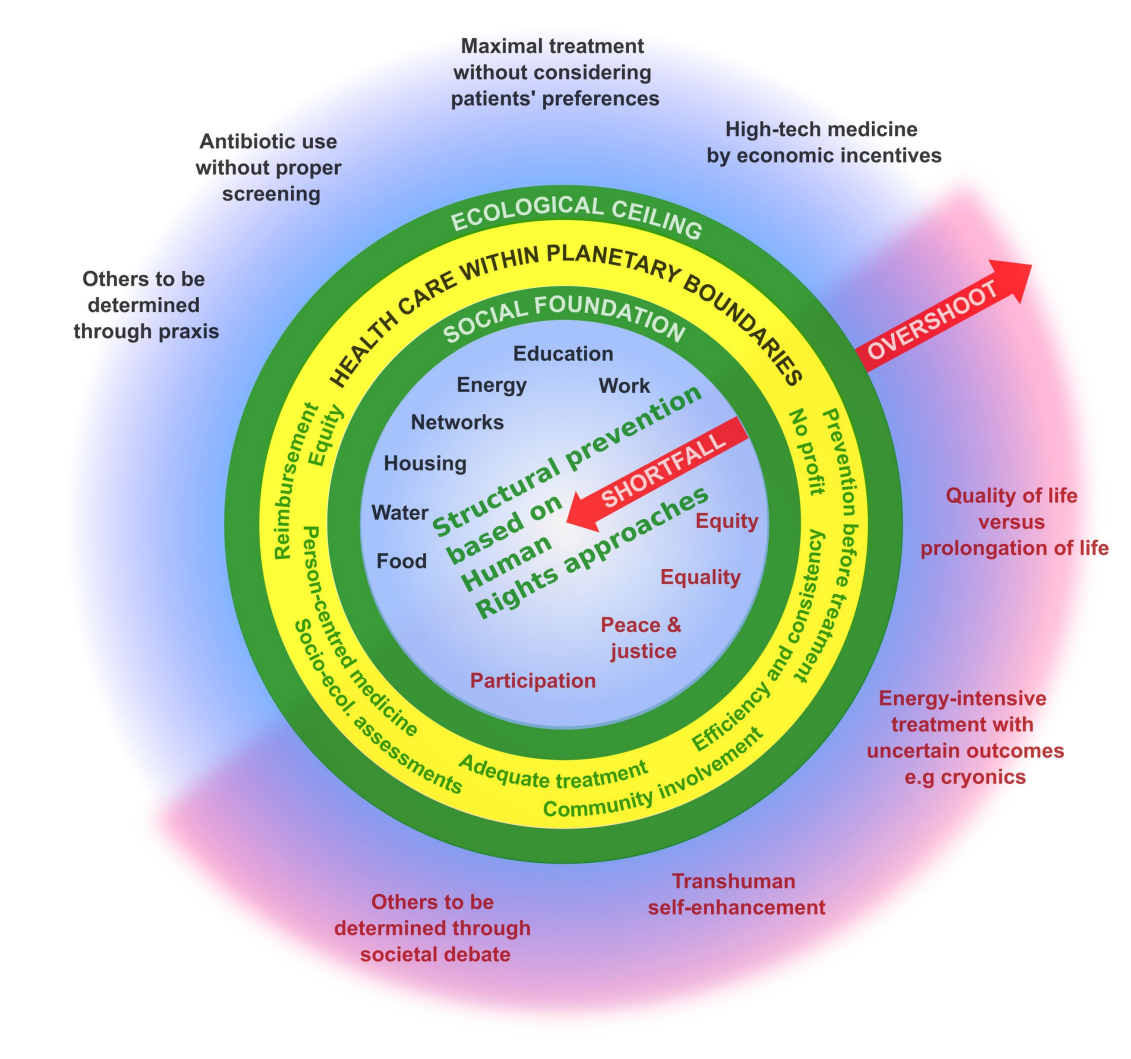
Health sufficiency framework. The figure shows orientations for health care within planetary boundaries inside the yellow ring. Based on the doughnut economic framework, they lie between a necessary social foundation that ensures good health care for all and the ecological ceiling that should not be exceeded by human resource use and environmental pollution. While the black features identify largely consensual aspects, the red ones within the inner circle indicate determinants of health that are not widely accepted or are often neglected. The red features in the outer circle indicate treatment options and practices that require societal debate.

Thus, it is necessary to discuss the concept of the *Health Sufficiency Framework*, which is illustrated in Figure 1. To be clear: *sufficiency* is about considering how better health and well-being can be achieved alongside the delivery of high-quality healthcare by diminishing shortfalls and avoiding overshoots (7, 8). Health sufficiency does not mean that patients have to compromise on the quality of healthcare, but that prevention is comprehensively emphasized. This requires a focus on *structural prevention*, which requires a transformation of living conditions as determined by many sectors, including transport, land use and urban planning, as well as agriculture and others. The aim is to address the wider social and structural determinants of health and to create enabling environments for better health and well-being for all, while ensuring there is a much lower environmental impact.

## Staying in the safe and just zone of humanity

Based on the doughnut economic model (9), health sufficiency is about bringing healthcare into a safe operating space within planetary limits (Figure 1), whereby planetary boundaries are not exceeded and all people have their basic needs met. To achieve this, it is necessary to *diminish shortfalls* in healthcare – that is, conditions under which basic prerequisites for a healthy life are not given – as well as *avoid overshoots* – that is, avoid treatments that are highly resource-and energy-intensive or environmentally polluting and do not provide significant health benefits. Figure 1 illustrates that these two realms (i.e., diminishing shortfalls and avoiding overshoots) have two parts. While the upper left portion describes consensual aspects, the lower right, comprises controversial issues that we suggest require wider political and societal discussion.

### Diminishing shortfalls

A human rights approach should be followed to ensure basic healthcare for all, and this includes developing healthy living conditions (i.e., structural prevention) (10). Commonly accepted determinants of health include education, income and working conditions; social networks; and housing; as well as access to electricity, clean water and safe sanitation; healthy food; and active modes of transport (11). Other factors are often not regarded as social determinants of health, including equity and equality, mental health, peace, justice and political participation.

### Avoiding overshoots

It is possible to identify some unquestionable overshoots, such as the use of maximal treatment without discussing this with the patient or the overuse of high-tech medicine that is incentivized by economic interests (12). The use of antibiotics without indication can be an overshoot when it occurs in high-resource healthcare settings that have appropriate diagnostic possibilities and sufficient hygiene standards (13–15). In the process of reorientation towards a health system that focuses on prevention, other potential overshoots may successively be determined. Moreover, it remains controversial whether certain practices represent overshoots, and these require discussion. These issues may include transhuman self-enhancement, energy-intensive treatments that have uncertain outcomes (e.g., cryonics) and others that need to be identified – often on a case-by-case basis (16–18).

## Reorienting towards healthcare within planetary boundaries

To maintain healthcare within planetary boundaries, we suggest three main areas of action: *reducing carbon footprints, use of resources and environmental damage; reorienting health systems’ objectives;* and *rethinking healthcare economics*.

### Reducing carbon footprints, use of resources and environmental damage

#### Adequate treatment

Adequate healthcare requires appropriate diagnostics, comprehensive evaluations, including a thorough medical history, and reflection on the individual health benefits. It is estimated that globally around 1 in 10 patients is harmed during healthcare, that more than 50% of this harm could be prevented, and 30% of healthcare provided is of low value (19, 20). 8 Million people die annually from poor quality care (21). Among the common causes of harm to patients are medication errors; unsafe surgical procedures; healthcare-associated infections; overworked medical staff; diagnostic errors, and lack of safe water, sanitation and hygiene (also known as WASH) services in healthcare facilities among others. Globally, one in five healthcare facilities lacks basic water, one in two lacks basic hand hygiene; one in ten has no sanitation, and, one billion people access healthcare facilities without reliable electricity or without electricity access at all (22). As the possibility of medical harm is omnipresent, careful consideration of medical interventions should be a core element of every physician’s mindset, and hence, when action is not urgently required, less burdensome and less resource-intensive solutions should be tried first to avoid overdiagnosis and overtreatment. However, great care needs to be taken not to delay therapies in cases in which early treatment can be life-saving or maintain the patient’s quality of life (e.g., stroke) or determine the course of a disease and its outcomes (e.g., cancer) (20, 23–25).

#### Efficiency and consistency

Healthcare practices should become more energy-and resource-efficient by implementing resource-sparing solutions and more sustainable supply chains, as well as through the use of more sustainable products and technologies. All technologies, both new and old, should be used consistently with the goal of reducing their environmental impacts. The circular economy needs to be a leading principle in the development of new materials and medical products. There is a need to facilitate research into sustainable solutions for healthcare, including through collaboration across disciplines and sectors, and to provide incentives for their implementation (26, 27).

### Reorienting health systems’ objectives

#### Prevention before treatment

The discussion of whether we indeed practise “healthcare” or “sick care” points out that the health system largely focuses on the treatment of illnesses. Instead, the focus should be placed on health promotion and the primary prevention of disease. To some extent, this embraces behavioral prevention – for example, by fostering health literacy and subsequently communicating about and promoting healthy choices and healthy lifestyles. However, we advocate for a greater emphasis on *structural prevention* that focuses on overall living conditions and the social, economic and environmental determinants of health (28). This would allow for synergies among disease prevention, health promotion and environmental protection, while clearly reaching into other arenas of change and towards other sectors (e.g., transport, nutrition and agriculture) (29). Structural prevention has great potential to prevent disease and induce multiple benefits (30), and it has proven to be highly cost effective, provided that all costs and benefits are considered (31, 32).

#### Person-centred medicine

Putting patients at the centre of healthcare allows for more individualised treatment. When used during consultations with patients, concepts such as *shared decision-making* and *narrative medicine* begin by acknowledging the specific individual life course of each patient, and their perceptions, understanding and individual life plans. The focus is on the relationship between a physician and a patient, with an intensified emphasis on medical counselling. These concepts also imply adjusting the legal regulation of doctor–patient responsibility to allow for more individualised treatment. For example, it is interesting how *informed consent* can be made legally secure for both parties, especially when patients and doctors jointly decide on an alternative or experimental treatment (33, 34).

The concept of *evidence-based medicine* is a fundamental principle of safe and reliable clinical treatment. However, not all treatment options have been evaluated or can be evaluated in randomized controlled trials. Many patient factors (e.g., female sex and certain age groups) have been historically underrepresented, and some variables cannot be included for methodological reasons (e.g., different approaches to the doctor–patient relationship). Thus, the findings of evidence-based medicine should not be the sole orientation for treatment choice, but treatment should specifically reflect patients’ values and unique circumstances (24, 35, 36).

Person-centred care is particularly important as climate change presents healthcare systems with new tasks and challenges, for example physically due to extreme weather events (37), as well as mentally, such as increasing climate and eco-anxiety. A recent global survey reported that 45% of youth had negative feelings about climate change that affected their daily life and functioning (38). In addition, a 2022 WHO report highlighted that climate change poses a direct risk to mental health by increasing exposure to natural disasters and damage of natural resources, while also worsening the impact of environmental, social, and economic determinants on mental well-being (39). Healthcare systems are inadequately prepared for these changes. New approaches to addressing these challenges, such as the “climate consultation” in general practice, are time-consuming and no remuneration system has yet been established (40, 41).

#### More community involvement in healthcare

Countries that deal with conditions of scarcity, such as low-and middle-income countries, prove the benefits of community-oriented health services on a daily basis (e.g., through the efforts of community health workers or the delivery of home-based care). Accordingly, urban neighbourhood management, social meeting spaces and spiritual and religious welfare can be understood as health promoting opportunities contributing to the *structural prevention* of illness. Communities should be encouraged to take responsibility for their own health by developing consciousness about planetary interconnections, and to account for vulnerabilities, which will ultimately empower them and create resilience (31, 42, 43).

### Rethinking healthcare economics

#### Turning away from profit in healthcare

Health systems should be regarded as common goods and not be solely driven by economic interests. Some economic incentives may be justified to promote efficiency among healthcare providers; however, healthcare providers should not operate on a principle of profit maximisation but rather on maximising health benefits and patients’ well-being. Decades of research show that for-profit healthcare does not outperform public or non-profit healthcare in terms of access, quality of care, or efficiency (44–47).

#### Implementing socio-ecological assessments

The health sector should be evaluated not only on financial costs and benefits but also on its ecological costs, as well as the benefits gained through environmental protection. Tools such as the Planetary Health Rapid Impact Assessment can be used for informed decision-making, and focus on sustainable policy outcomes to promote sufficiency in healthcare and beyond (48).

#### Reorganising reimbursement

Whether financing hospitals and physicians through pay-for-performance or bonus schemes improves health care strongly depends on the design of the scheme and the context of its application. It has been shown that these concepts may provide false incentives and that instead other motivations and attitudes of physicians better improve treatment quality (49–52).

Ensuring that healthcare is more person-oriented implies rethinking medical remuneration for health services and honouring intensified relations between healthcare workers and patients. Such approaches are time intensive, and since most of the time spent is not reimbursed, they are currently not economically viable. Moreover, as benefits in terms of disease prevention are not obvious and can hardly be directly observed, patient-centred medicine is often not the norm in conventional medicine.

## Equity orientation

A guiding principle for the many decisions that must be taken on the path towards delivering healthcare within planetary boundaries is *equity orientation*. Medical treatment should focus on preserving people’s health status to enable them to have a good life, and it should be provided for all, on an equal, non-discriminatory basis. The leading question should be justice oriented, in the sense of: who needs what for achieving an equal chance of living a healthy life? The fulfilment of health-related basic human rights, such as the right to food and access to safe water and adequate sanitation, are non-negotiable minimum requirements (53). Furthermore, other substantive elements of the human right to a clean, healthy, and sustainable environment – including clean air and non-toxic environments in which to live, work, study, and play – need to be recognized as integral components of public health policy and prioritised, allowing all people to live a life of dignity (54). Today, these topics should always be addressed while also considering approaches to climate and environmental justice (18, 55, 56).

The question of equity can fuel a societal debate about alternative notions of well-being and healthcare: What does “best health” mean? Can it be achieved only through more health services, more medication, more high-tech treatment options? How healthy do I need to be to live a fulfilled and meaningful life? Is the imperative of bodily recovery to be favoured over aspects of quality of life?

## Fundamental philosophical reflection is needed

Health sufficiency requires intensified reflection about basic aspects of human life. Human lives are finite, at least this is the momentary status quo. All living creatures are subject to natural ageing. At the same time, technology allows humans to push our natural boundaries further and to optimize, for example, our physical performance in sports, our intellectual performance, or our beauty. What is an appropriate way of dealing with these facts? Beauty also affects the human quality of life and is increasingly made a matter of financial choice. Should transhuman attempts at self-enhancement and the prolongation of life even be feasible? For example, should cryonics be allowed to attempt to overcome the all-too-human judgement of death for a financially capable person until later societies have the technology to cure the diseases that afflict that person? Is cryonics sheer arrogance *per se* or does the fact that this treatment requires tremendous energy consumption make a difference? Currently, self-enhancement technologies attract many people with their promises of improved personal performance, whether physically in sports or intellectually by strengthening concentration. The temptation is obvious, but are there also possible losses? (57).

We have deliberately chosen some of the more distinctive technological examples as we believe they give a good indication of the direction our civilisations are taking towards overuse—also in healthcare. One example for the healthcare setting is the use of advanced diagnostic imaging technologies, that while certainly improving diagnostic accuracy, outcomes and treatments in many cases—also have some rebound effects leading to a large number of diagnostic imaging performed with potential adverse effects for patients alongside large energy and resource use (58). There is evidence that globally a considerable amount of diagnostic imaging is unnecessary and inappropriate (59). To be clear: technological innovation is a critical enabler for climate and environmental action and one part of the required system change (60). Universal access to clean energy and technologies will reduce pollution, and improve air quality, health and equity (61). Technological innovation can however lead to new and greater environmental impacts and rebound effects that can increase health risks and energy needs, alongside increasing social inequalities and overdependence on technological knowledge and technology providers (60, 62, 63).

## Living life to its very essence

With regard to the global environmental crisis, it is often argued that indigenous communities possess special wisdom that might be needed today. As they have adapted to nature from time immemorial, they might also contribute to solving global environmental problems. In debates about Planetary Health, a planetary consciousness is considered necessary, which indigenous peoples who live in proximity to nature have never lost. However, learning from these communities would likely require a reorientation in thinking about health and pain and the inevitabilities of life. Traditional healthcare is less about healing bodily symptoms and more about harmonising a patient’s spiritual and social life (64). While we should continue our best efforts to reduce the burden of disease through research, prevention and adequate treatment, we should not ignore that humans are natural beings in natural cycles of life and death, a reality that often seems to be forgotten in health-related discourse and sometimes also in healthcare. These considerations include issues of respecting patients’ wishes at the end of their life. The vulnerability of terminally ill patients in hospitals and retirement homes became evident during the COVID pandemic, when they were not allowed to have family visits during their last hours. A related example is the inherent preference of many healthcare systems for prolonging life, even for patients who have an advanced healthcare directive stating other wishes. This may be partly due to legal concerns and to the difficulty of immediately determining the reliability of patients’ wishes in emergency situations.

How can modern society secure dignity for people who suffer, live with chronic diseases or are in the process of dying, when at the same time individual performance is the most appreciated value of economics? The answers to these questions are not easy. In such controversial cases, consensus is unlikely to be reached. However, discussing such natural issues of the human condition and a good life is vital for societies to help them adjust their practices and policies to the necessities of a rapidly changing world.

## Health sufficiency summarised

Changing healthcare to conform to planetary boundaries requires more than pushing a few buttons or adjusting a few knobs. Social change for a sustainable future requires making profound changes in the orientation and organization of healthcare as well as the political regulation of general living conditions and individual lifestyle options. It further requires consideration of deeply consolidated common beliefs and value systems. Discussions around changes in healthcare services relate to individual experiences and preferences and often prove to be controversial. However, given the destructive impact on health and well-being caused by the increasingly frequent crossing of planetary boundaries, fundamental change that surpasses surface-level technological solutions is necessary, especially in high-income countries. Political and public debates about the right way to move forward to achieve healthcare within planetary boundaries are, thus, very necessary. Planetary Health is evidently a political question of how to provide the best and most equitable healthcare for all within the limits of the planet.

The fundamental question of health sufficiency is: What does a person and societies really need to be able to live well? With this focus on the very essence of human life, the question might even become the point of reference for many public and political debates about a variety of questions relating to the great socio-ecological transformation needed to ensure Planetary Health. Health is the highest good for communities and for each person individually.

## Data Availability

The original contributions presented in the study are included in the article/supplementary material, further inquiries can be directed to the corresponding author.
